# Supplementary material for: Nucleotide mismatches prevent intrinsic self-silencing of hpRNA transgenes to enhance RNAi stability in plants
Source: Nat Commun. 2022 Jul 7;13:3926. doi: 10.1038/s41467-022-31641-5 (PMC9263138; doi:10.1038/s41467-022-31641-5)
Supplement: Supplementary file 1 — Supplementary Information [file 41467_2022_31641_MOESM1_ESM.pdf]

Nucleotide mismatches prevent intrinsic self-silencing of hpRNA transgenes to enhance RNAi stability in plants

Zhang *et al.*

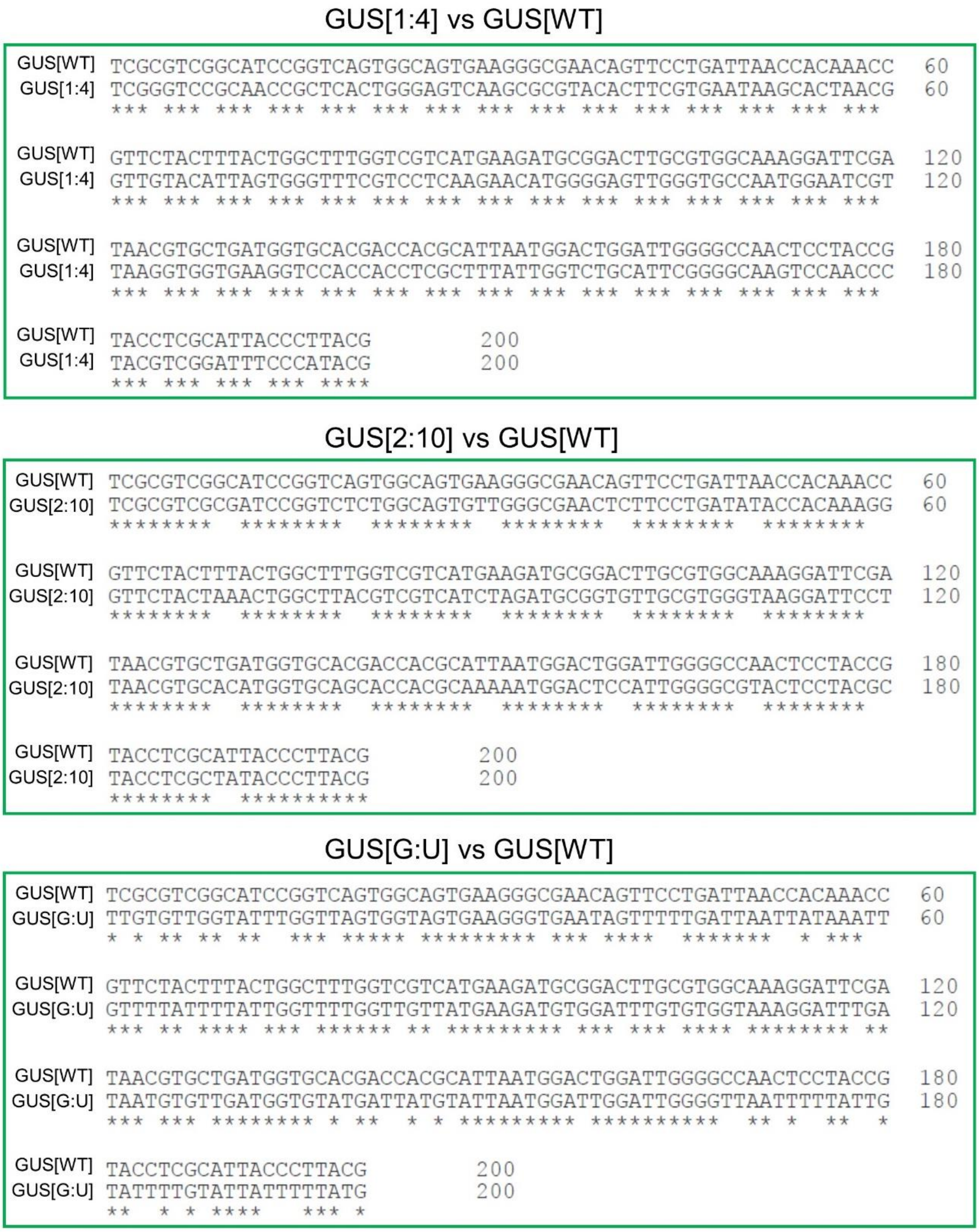

Supplementary Figure 1. **Sequence alignment between modified *GUS* sequences with wild-type *GUS* sequence.** The asterisks indicate the unchanged nucleotides in the modified GUS[1:4] (top), GUS[2;10] (middle) and GUS[G:U] (bottom) sequences.

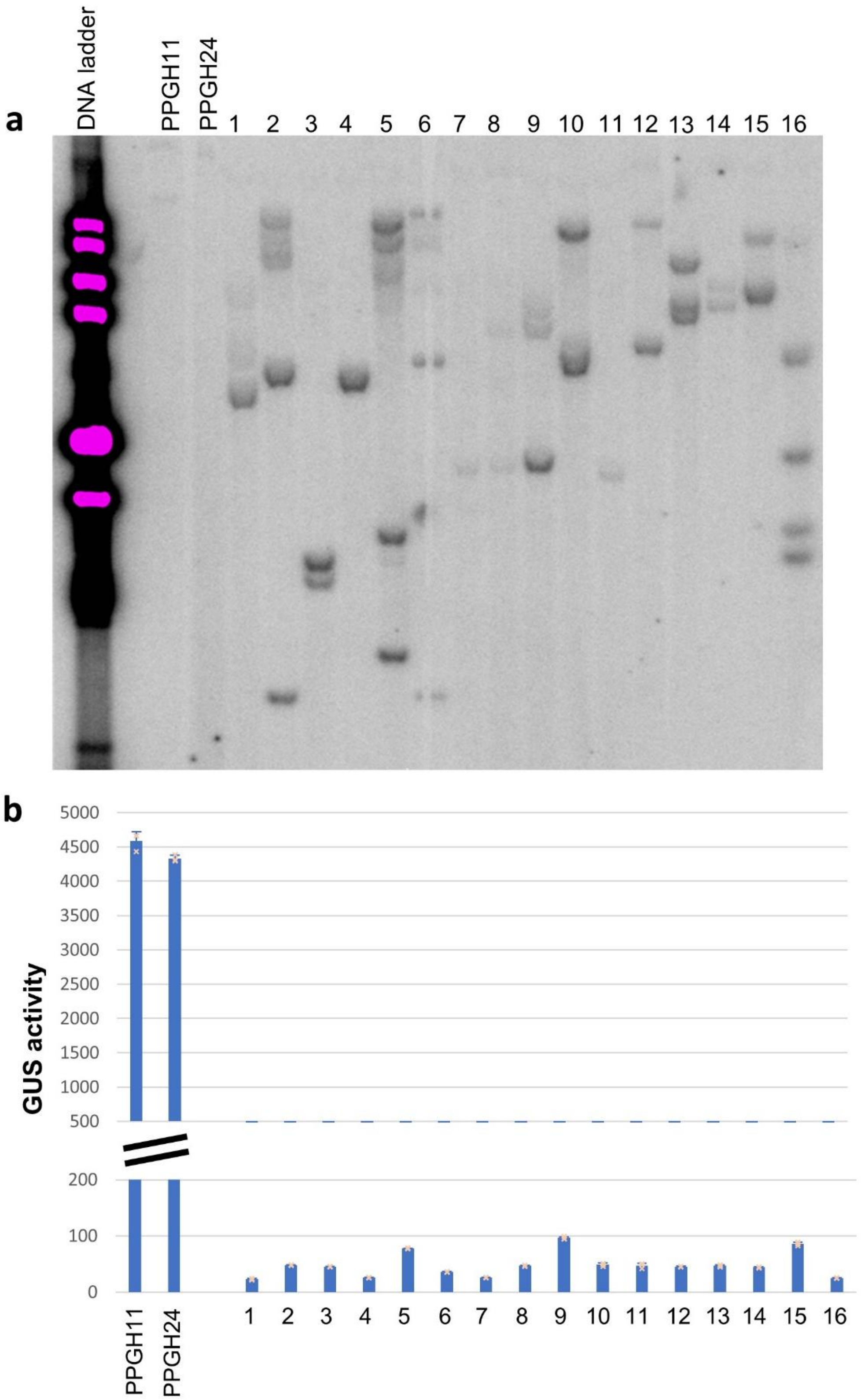

Supplementary Figure 2. Southern blot hybridization shows variable transgene insertions in sixteen independent hpGUS[G:U] transgenic lines. a Southern blot hybridization of hpGUS[G:U] transgenic lines. DNA was digested with *Hin*dIII prior to gel electrophoresis and probed with an OCS-T probe. **b** GUS expression levels of the T1 transgenic lines analysed. Three technical replicates were measured for each line, and data are presented as mean values +/- s.d. with the three data points shown as orange crosses. Source data are provided as a Source Data file.

EIN2[G:U] vs EIN2[WT]   (43 of 200 C to T conversion)

```
EIN2[WT]  GACCTCAGCTAGGGTTTATCCAGAGAATGGTTCCTGCTCTACTTCCTGTCCTTTTGGTTT   60
EIN2[G:U] GATTTTAGTTAGGGTTTATTTAGAGAATGGTTTTTGTTTTATTTTTTGTTTTTTTGGTTT   60
          **  * ** **********  ***********  ** * ** **  ***  *********

EIN2[WT]  CTGTCGGATATATTGATCCCGGGAAATGGGTTGCAAATATCGAAGGAGGTGCTCGTTTCG   120
EIN2[G:U] TTGTTGGATATATTGATTTTGGGAAATGGGTTGTAAATATTGAAGGAGGTGTTTGTTTTG   120
           *** ************   ************* ****** ********** * **** *

EIN2[WT]  GGTATGACTTGGTGGCAATTACTCTGCTTTTCAATTTTGCCGCCATCTTATGCCAATATG   180
EIN2[G:U] GGTATGATTTGGTGGTAATTATTTTGTTTTTTAATTTTGTTGTTATTTTATGTTAATATG   180
          ******* ******* ***** * ** **** *******  *  ** *****  ******

EIN2[WT]  TTGCAGCTCGCATAAGCGTT 200
EIN2[G:U] TTGTAGTTTGTATAAGTGTT 200
          *** ** * * ***** ***
```

PDS[G:U] vs PDS[WT]   (82 of 450 C to T conversion)

```
PDS[WT]   GAAAATAAAGTTTGCTATTGGACTTTTGCCAGCCATGGTCGGCGGTCAGGCTTATGTTGA   60
PDS[G:U]  GAAAATAAAGTTTGTTATTGGATTTTTGTTAGTTATGGTTGGTGGTTAGGTTTATGTTGA   60
          ************** ******* *****  **  ***** ** *** *** *********

PDS[WT]   GGCCCAAGATGGTTTATCAGTCAAAGAATGGATGGAAAAGCAGGGAGTACCTGAGCGCGT   120
EIN2[G:U] GGTTTAAGATGGTTTATTAGTTAAAGAATGGATGGAAAAGTAGGGAGTATTTGAGTGTGT   120
          **   ************ *** ****************** ********  **** * **

PDS[WT]   GACCGACGAGGTGTTTATTGCCATGTCAAAGGCGCTAAACTTTATAAACCCTGATGAACT   180
PDS[G:U]  GATTGATGAGGTGTTTATTGTTATGTTAAAGGTGTTAAATTTTATAAATTTTGATGAATT   180
          **  ** *************  **** ***** * **** ********   ******* *

PDS[WT]   GTCAATGCAATGCATTTTGATAGCTTTGAACCGGTTTCTTCAGGAAAAACATGGTTCCAA   240
PDS[G:U]  GTTAATGTAATGTATTTTGATAGTTTTGAATTGGTTTTTTTAGGAAAAATATGGTTTTAA   240
          ** **** **** ********** ******  ***** ** ******** ******  **

PDS[WT]   GATGGCATTCTTGGATGGTAATCCTCCGGAAAGGCTTTGTATGCCAGTAGTGGATCATAT   300
PDS[G:U]  GATGGTATTTTTGGATGGTAATTTTTTGGAAAGGTTTTGTATGTTAGTAGTGGATTATAT   300
          ***** *** ************  *  ******* ********  ********** ****

PDS[WT]   TCGATCACTAGGTGGGGAAGTGCAACTTAATTCTAGGATAAAGAAAATTGAGCTCAATGA   360
EIN2[G:U] TTGATTATTAGGTGGGGAAGTGTAATTTAATTTTAGGATAAAGAAAATTGAGTTTAATGA   360
          * *** * ************** ** ****** ******************* * *****

PDS[WT]   CGATGGCACGGTTAAGAGTTTCTTACTCACTAATGGAAGCACTGTCGAAGGAGACGCTTA   420
PDS[G:U]  TGATGGTATGGTTAAGAGTTTTTTATTTATTAATGGAAGTATTGTTGAAGGAGATGTTTA   420
           ***** * ************ *** * * ********* * *** ******** * ***

PDS[WT]   TGTGTTTGCCGCTCCAGTCGATATCCTGAA 450
PDS[G:U]  TGTGTTTGTTGTTTTAGTTGATATTTTGAA 450
          ********  * *  *** *****  ****
```

Supplementary Figure 3. **Sequence alignments between G:U modified and WT sequences of *EIN2* and *PDS*.** The asterisks indicate the unchanged nucleotides in the modified EIN2[G:U] (top) and PDS[G:U] (bottom) sequences.

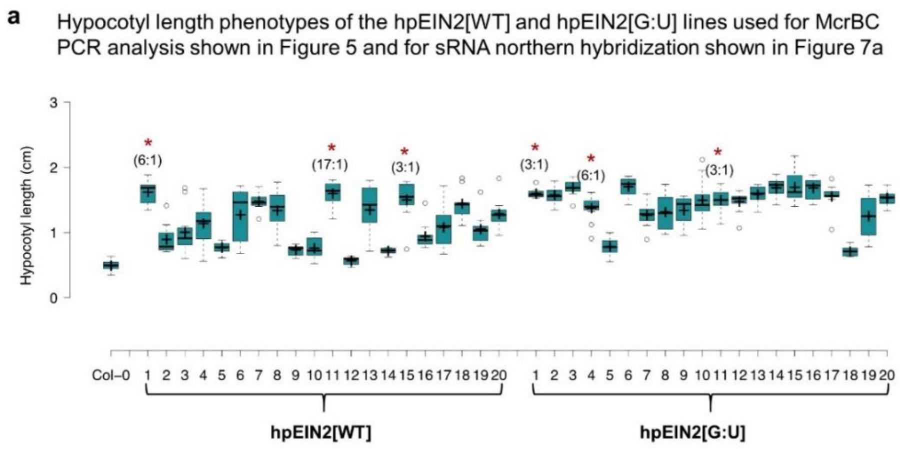

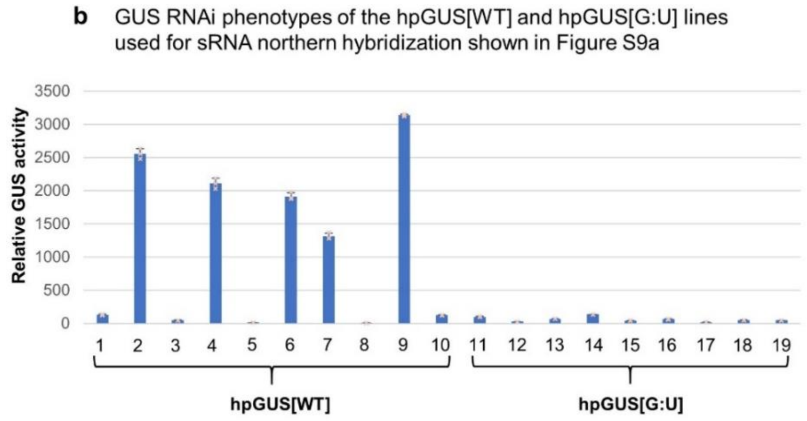

Supplementary Figure 4. **RNAi phenotypes of hpEIN2 and hpGUS lines used in Figures 5, 7a, and Supplementary Figure 9a, respectively. a** Hypocotyl length of the 20 independent lines each of hpEIN2[WT] and hpEIN2[G:U] shown in Figure 2, including the 12 lines each analysed by McrBC-digestion PCR in Figure 5a, and the 10 hpEIN2[WT] and 9 hpEIN2[G:U] lines used for sRNA northern blot hybridization in Figure 7a. The central horizontal line indicates the median value, the lower and upper borders of the box represent the first and third quartiles as determined by R software (BoxPlotR, http://shiny.chemgrid.org/boxplotr/); whiskers extend 1.5 times the interquartile range from the first and third quartiles; outliers are represented by dots; crosses represent sample means. n = 32, 12, 11, 12, 12, 12, 11, 10, 11, 10, 10, 10, 10, 10, 10, 10, 10, 10, 10, 10, 10, 12, 11, 12, 11, 11, 10, 11, 11, 11, 12, 12, 12, 12, 10, 12, 10, 10, 12, 12, 12 sample points, respectively. The red asterisks indicate lines that were further analysed using bisulfite sequencing in Figure 5b. The numbers in bracket indicate the kanamycin resistance:sensitivity ratios of segregating T2 siblings for the specific line. **b** *GUS* RNAi phenotypes of the 19 independent hpGUS[WT] and hpGUS[G:U] lines used for sRNA northern blot hybridization in Supplementary Figure 9a. Three technical replicates were measured for each line, and data are presented as mean values +/- s.d. with the three data points shown as orange crosses. Source data are provided as a Source Data file.

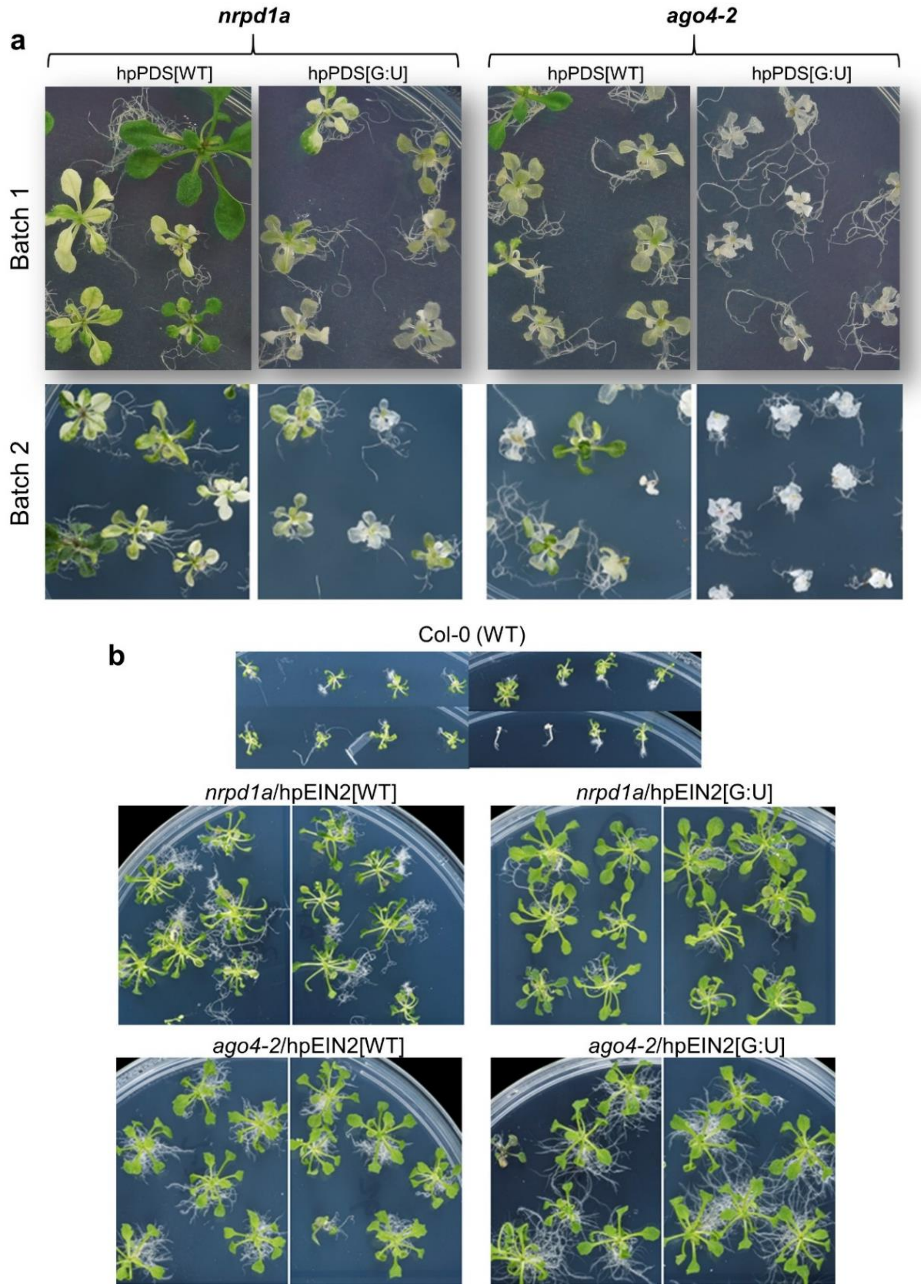

Supplementary Figure 5. **RNAi phenotypes in primary (T1) hpPDS[WT] and hpPDS[G:U] lines (a) and in T2 hpEIN2[WT] and hpEIN2[G:U] plants (b) of the *nrpd1a-3* and *ago4-2* mutant backgrounds.** For hpPDS, each plant is an independent transgenic line; for hpEIN2, plants inside each rectangle represent siblings of an independent line. *EIN2* RNAi was assayed under light on ACC medium because many of the T2 *ago4-2*/hpEIN2[G:U] seed showed slow and low rate of germination in the dark. The hpEIN2[G:U] plants showed stronger growth than the hpEIN2[WT] plants in both mutant backgrounds, indicating stronger *EIN2* RNAi. The *ago4-2*/hpEIN2[G:U] plants showed the most vigorous root growth indicating the strongest *EIN2* RNAi.

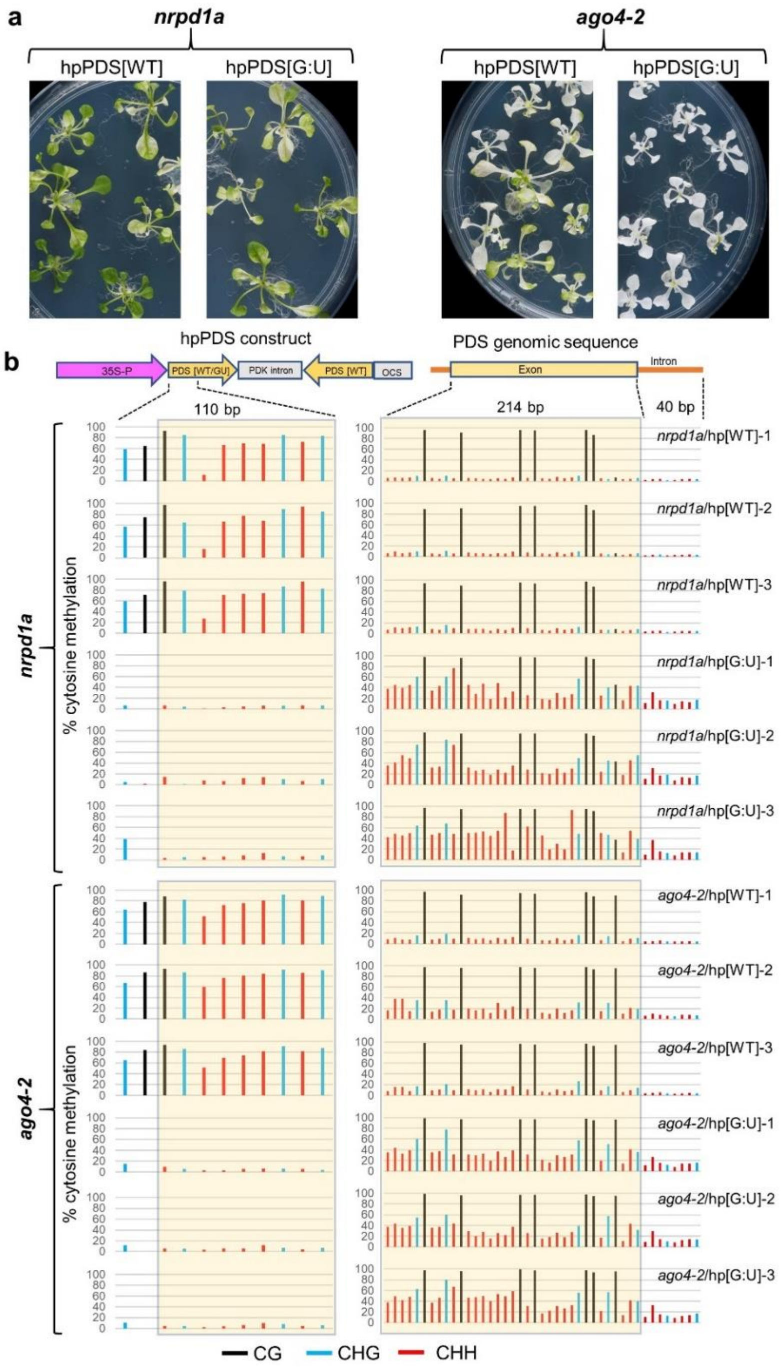

Supplementary Figure 6. **hpPDS[G:U] transgene induces stronger DNA methylation at the target *PDS* genomic sequence than hpPDS[WT] transgene in *nrpd1a-3* and *ago4-2* mutants. a** hpPDS[WT] induces uniform but weaker photobleaching phenotypes than hpPDS[G:U] in *nrpd1a-3* and *ago4-2*. The plants are primary T1 transgenics. Note the extreme photo bleaching of the *ago4-2*/hpPDS[G:U] plants indicating extreme *PDS* RNAi. **b** Bisulfite sequencing showing strong DNA methylation in the IR DNA of hpPDS[WT] transgene but weaker methylation at the *PDS* genomic target than in the hpPDS[G:U] plants (top DNA strand only). The yellow-highlighted areas represent the hpPDS IR (left) and the hpRNA-targeted PDS genomic sequence (right). The IR region of hpPDS[G:U] has no cytosines, so the signals represent background noises. Source data are provided as a Source Data file.

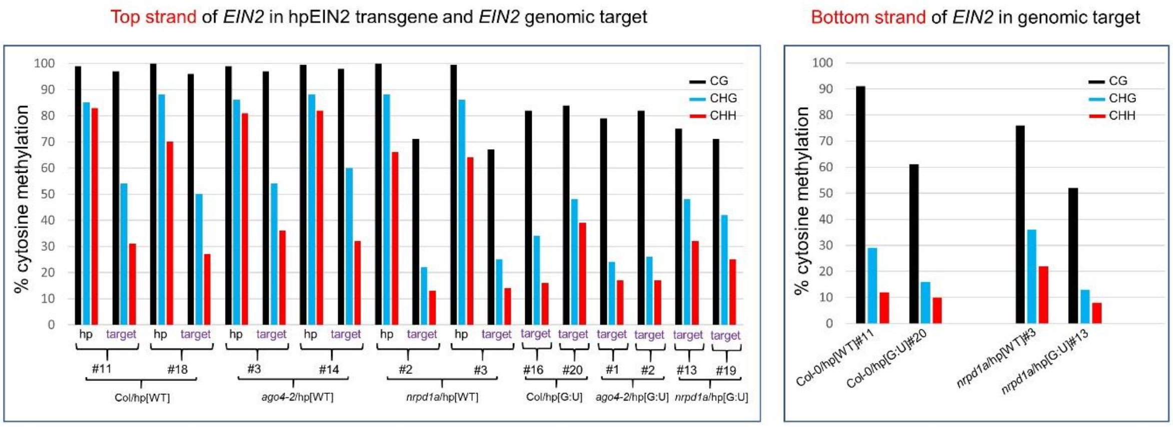

Supplementary Figure 7. **Average levels of IR and target *EIN2* gene methylation in the hpEIN2[WT] and hpEIN2[G:U] lines shown in Figure 6 (top strand only) and average methylation levels of the bottom DNA strand of target *EIN2* gene shown in Supplementary Figure 8.** Note the reduction in target sequence methylation in *nrpd1a-3* compared to Col-0 backgrounds for the hpEIN2[WT] but not the hpEIN2[G:U] lines (left panels). Also note the strong reduction of methylation in the bottom strand compared to the top strand of *EIN2* target gene in the Col/hpEIN2[G:U]#20 and *nrpd1a*/hpEIN2[G:U]#13 lines (compare left with right panels). Source data are provided as a Source Data file.

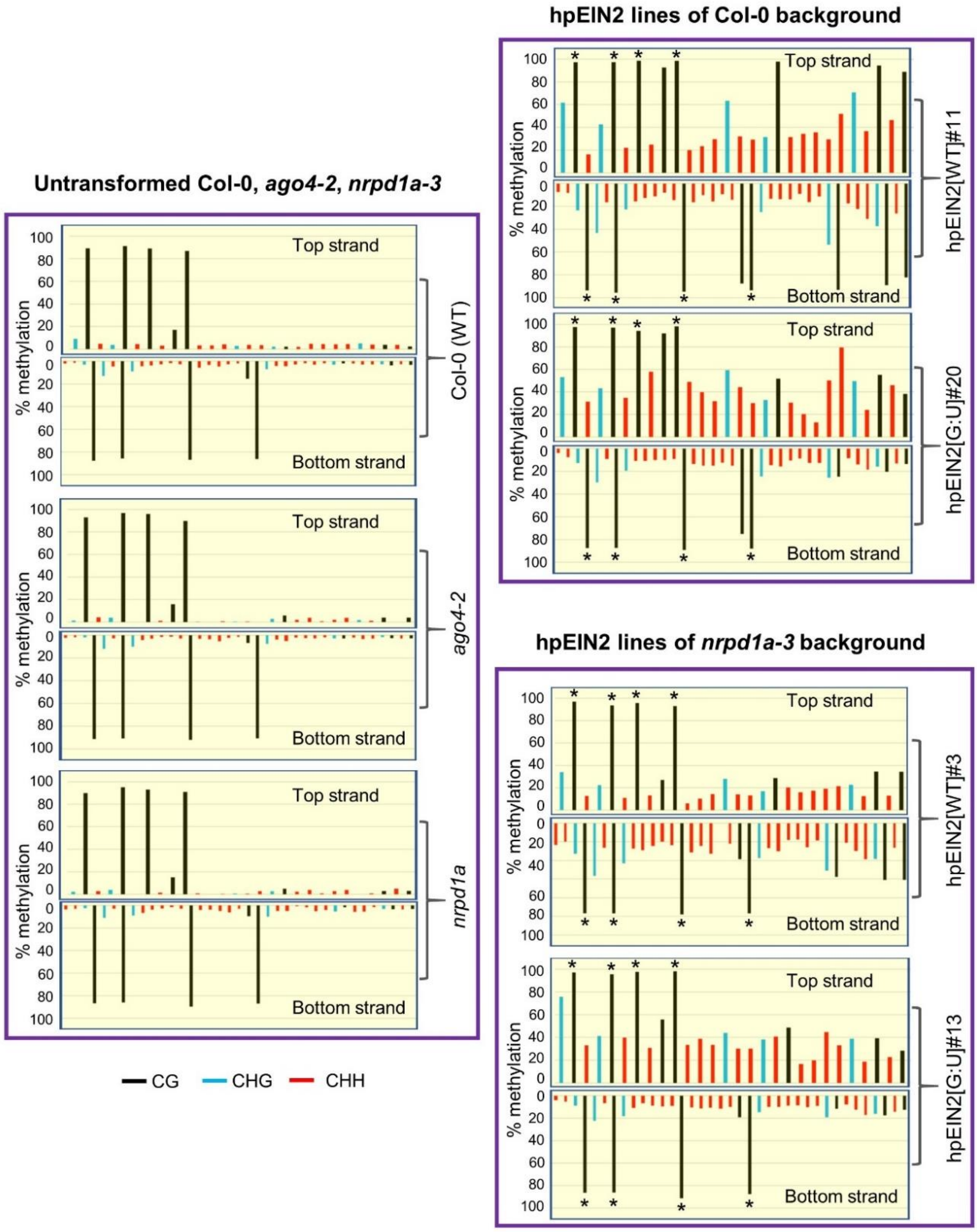

Supplementary Figure 8. The bottom strand of the *EIN2* target gene shows much reduced cytosine methylation compared to the top strand in the Col-0/hpEIN2[G:U] and *nrpd1a-3*/hpEIN2[G:U] plants. This is particularly true for the *nrpd1a-3*/hpEIN2[G:U] plant, where cytosines of all sequence contexts (except for the four CG sites indicated by asterisks that are already densely methylated in the untransformed plant) show greatly diminished methylation compared to the top strand. The hpEIN2[WT] plants, in contrast, showed no (the *nrpd1a-3* background) or reduced (the Col-0 background) strand bias in target *EIN2* gene methylation. This result suggests that RdDM requires strong sequence complementarity between siRNAs and target DNA strands, and that siRNAs from the sense strand of hpEIN2[G:U] are inefficient at inducing RdDM due to T:C sequence mismatches with the target DNA strand, namely the bottom strand of *EIN2*. Source data are provided as a Source Data file.

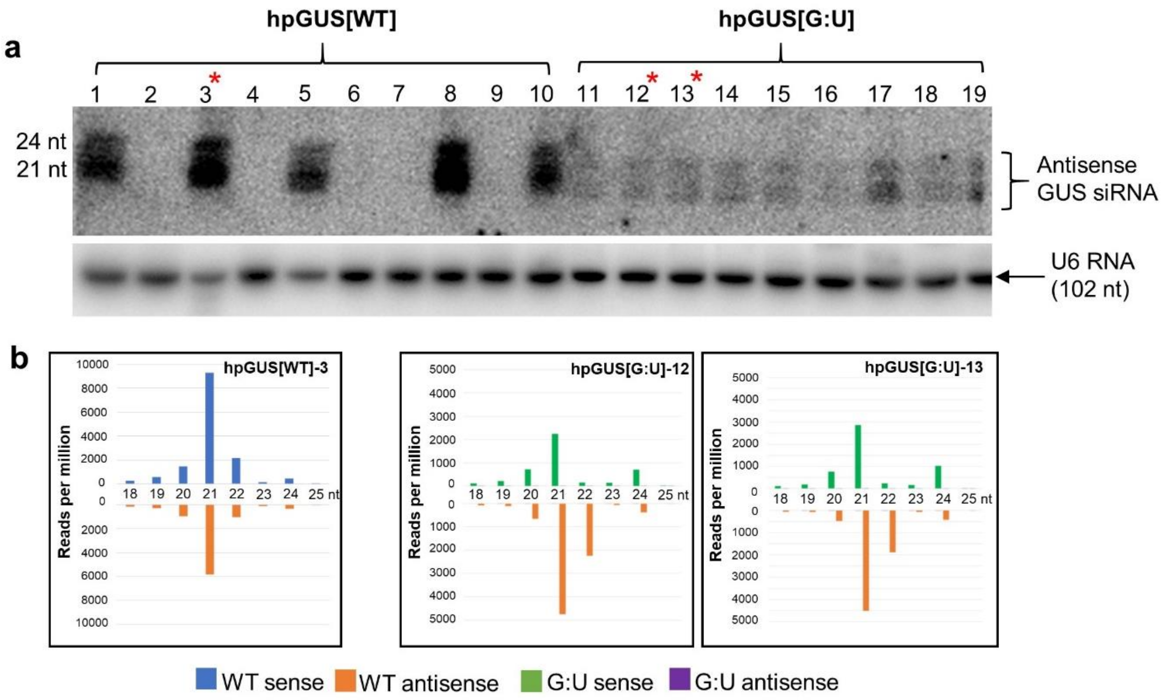

Supplementary Figure 9. **siRNA analysis of hpGUS[WT] and hpGUS[G:U] plants. a** Northern blot hybridization to detect antisense siRNAs in T0 hpGUS[WT] and hpGUS[G:U] lines using the 200 bp sense *GUS* target sequence as probe. **b** Summary of sRNA deep sequencing data of T0 hpGUS[WT] and hpGUS[G:U] lines. Source data are provided as a Source Data file.

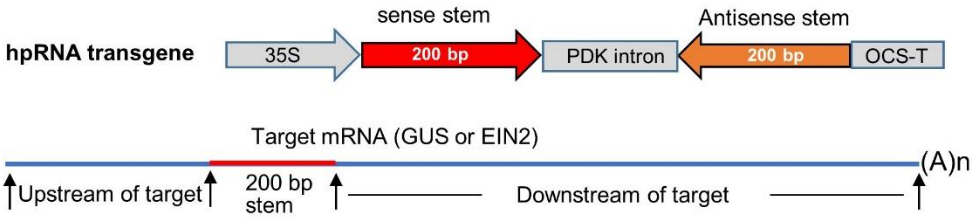

1st sRNA sequencing

Construct	Total No. 20-24 nt siRNAs (per million 18-30 nt reads)				
	35S	Sense stem	Antisense stem	PDK intron	Downstream of target
hpEIN2[WT]-7	21	7369	13802	11	12
hpEIN2[WT]-9	59	9032	6513	31	13
hpEIN2[G:U]-14	84	14909	3033	191	39
hpEIN2[G:U]-15	59	25443	3996	258	38
hpGUS[WT]-3	6	13404	8232	2	100
hpGUS[G:U]-12	2	3977	8152	1	17
hpGUS[G:U]-13	1	5029	7363	1	28

2nd sRNA sequencing

Line	Total No. 20-24 nt siRNAs (per million 18-30 nt reads)				
	35S	Sense stem	Antisense stem	PDK intron	Downstream of target
nrpd1a/hp[WT]-2	125	3336	3623	4	16
nrpd1a/hp[WT]-13	224	1485	1769	22	32
nrpd1a/hp[G:U]-2	817	8951	2084	100	35
nrpd1a/hp[G:U]-18	572	14214	4210	46	30
ago4-2/hp[WT]-10	354	29268	25679	193	4
ago4-2/hp[WT]-11	1211	50008	43570	310	10
ago4-2/hp[G:U]-4	11	91758	21967	306	3
ago4-2/hp[G:U]-6	335	159522	43035	553	6
Col/hp[WT]-11	255	18128	36527	56	24
Col/hp[G:U]-14	166	13194	3637	142	30
Col/hp[G:U]-20	98	22541	5056	296	33

Supplementary Figure 10. Summary of sRNA reads from the different regions of hpGUS and hpEIN2 transgenes and the respective target genes. The table on the top is the summary of the 1st deep sequencing data, and the table below is of the second deep sequencing data. For the bottom table, the plant lines (T2 population) of the Col-0 background correspond to the same lines in Figure 2b (hp[WT]-11, hp[G:U]-14, 20), and some (but not all) of the plant lines (T2) of the *ago4-2* and *nrpd1a-3* backgrounds correspond to the those in Figure 7b (*ago4-2*/hp[WT]-10, *ago4-2*/hp[G:U]-6, and *nrpd1a*/hp[WT]-2). There are almost no sRNA reads from the upstream region of the target mRNA so the numbers are not listed.

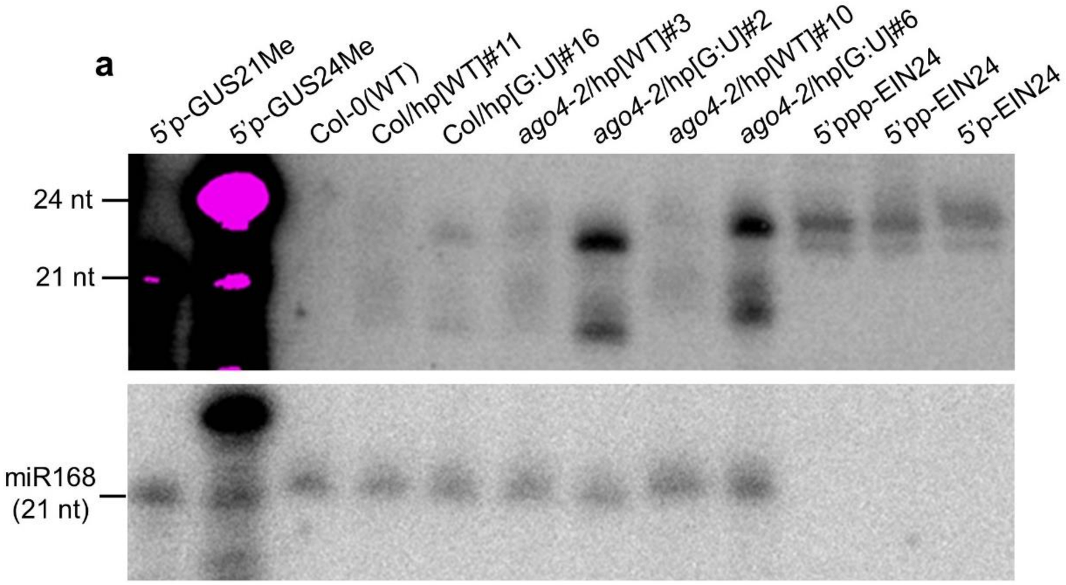

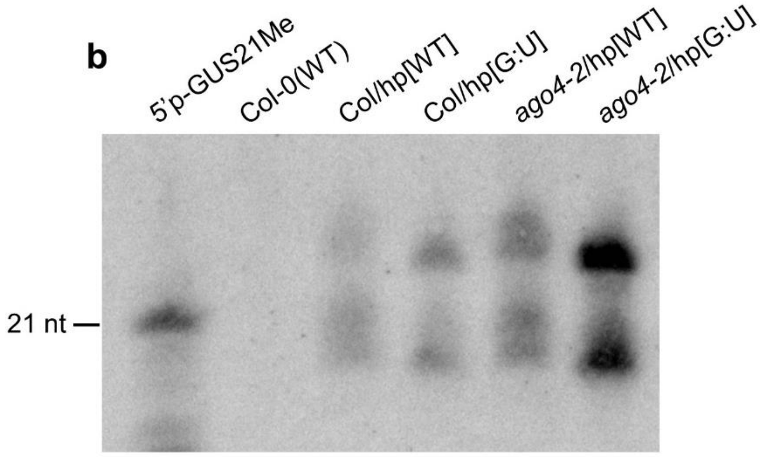

Supplementary Figure 11. **Northern blot hybridization analysis of hpEIN2[WT] and hpEIN2[G:U] plants additional to Figure 7e. a** Northern blot hybridization of hpEIN2[WT] and hpEIN2[G:U]-derived siRNAs together with 5' radioactively labelled 21 and 24 nt (5'p-GUS21Me, 5'p-GUS24Me) synthetic GUS sRNA markers (Supplementary Data 1; all with 3'-O-methyl or Me), and *in vitro* transcribed mono- (5'P), di- (5'PP) and tri- (5'PPP) phosphorylated *EIN2* sRNA markers (see Methods). These in vitro transcripts were visualized by hybridization with antisense oligonucleotide probe. The same blot was stripped and rehybridized with miR168 probe as loading control. Note that the G:U hpRNA-derived 24-nt siRNAs appeared to migrate the same as the tri- and di-phosphorylated markers. **b** Additional northern blot hybridization of the same five RNA samples for Figure 7e, with 5' radioactively labelled 21 nt synthetic GUS sRNA marker (5'p-GUS21Me). sRNA separation for both northern blots was performed in 17% urea-polyacrylamide gel (19:1 acrylamide:bis). Source data are provided as a Source Data file.

Supplementary Table 1. Primers for making hpGUS[WT], hpGUS[G:U], hpGUS[1:4] and hpGUS[2:10] constructs as well as hpEIN2[WT] and hpEIN2[G:U] constructs.

**Primer**	**Sequence**
GUS-WT-F	cctcgaggatccTCGCGTCGGCATCCGGTC
GUS-WT-R	gggtaccaagcttCGTAAGGGTAATGCGAGGTA
GUS-GU-F	ccctcgagTTGTGTTGGTATTTGGTTAGTGGTAGTGAAGGGTGAATAGTTTTTGATTAATTA TAAATTGTTTTATTTTATTGGTTTTGGTTGTTATGAAGATGTGGATTTGTGTGGTA
GUS-GU-R	ggggtaccCATAAAAATAATACAAAATACAATAAAAATTAACCCCAATCCAATCCATTAA TACATAATCATACACCATCAACACATTATCAAATCCTTTACCACACAAATCCACATCT
GUS-4M-F	ccctcgagTCGgGTCcGCAaCCGcTCAcTGGgAGTcAAGcGCGtACAcTTCgTGAaTAAgCACtAA CgGTTgTACaTTAgTGGgTTTcGTCcTCAaGAAcATGgGGAgTTGgGTGcCA
GUS-4M-R	ggggtaccgGTAtGGGaAATcCGAcGTAgGGTtGGAcTTGcCCCgAATgCAGaCCAaTAAaGCGa GGTgGTGgACCtTCAcCACcTTAaCGAtTCCaTTGgCACcCAAcTCCcCATgT
GUS-10M-F	ccctcgagTCGCGTCGcgATCCGGTCtcTGGCAGTGttGGGCGAACtcTTCCTGATatACCACAA AggGTTCTACTaaACTGGCTTacGTCGTCATctAGATGCGGtgTTGCGTGGgt
GUS-10M-R	ggggtaccgcTAAGGGTAtaGCGAGGTAgcGTAGGAGTacGCCCCAATggAGTCCATTttTGCGT GGTgcTGCACCATgtGCACGTTAagGAATCCTTacCCACGCAAcaCCGCATCT
EIN2wt-F	CCTCGAGGATCCTCTAGACCTCAGCTAGGGTTTATC
EIN2wt-R	GGGTACCAAGCTTAACGCTTATGCGAGCTGCAA
EIN2-GU-F	CCTCGAGTCTAGATTTAGTTAGGGTTTATTTAGAGAATGGTTTTTGTTTTATTTTTTGT TTTTTTGGTTTTTGTTGGATATATTATTTTGGGAAATGGGTTGTAAATATTGAAGGAG
EIN2-GU-R	GGGTACCAACACTTATACAAACTACAACATATTAACATAAAATAACAACAAAATTAA AAAACAAAATAATTACCACCAAATCATACCCAAAACAAACACCTCCTTCAATATTTAC AACC

Supplementary Table 2. DNA fragments of 450 bp wild-type and C-to-T converted sequence of *PDS* cDNA for making hpPDS constructs (bold, underlined letters are introduced nucleotides for restriction sites).

**DNA fragment**	**Fragment sequence**
PDS-WT	**CCTCGAGGGATCCGAATTCATCGAT**GAAAATAAAGTTTGCTATTGGACTTTTGCCAGCCATG<br>GTCGGCGGTCAGGCTTATGTTGAGGCCCAAGATGGTTTATCAGTCAAAGAATGGATGGAAAAG<br>CAGGGAGTACCTGAGCGCGTGACCGACGAGGTGTTTATTGCCATGTCAAAGGCGCTAAACTTTA<br>TAAACCCTGATGAACTGTCAATGCAATGCATTTTGATAGCTTTGAACCGGTTTCTTCAGGAAAA<br>ACATGGTTCCAAGATGGCATTCTTGGATGGTAATCCTCCGGAAAGGCTTTGTATGCCAGTAGTG<br>GATCATATTCGATCACTAGGTGGGGAAGTGCAACTTAATTCTAGGATAAAGAAAATTGAGCTCA<br>ATGACGATGGCACGGTTAAGAGTTTCTTACTCACTAATGGAAGCACTGTCGAAGGAGACGCTTA<br>TGTGTTTGCCGCTCCAGTCGATATCCTGAA**TCTAGAGAATTCAAGCTTGGTACCc**
PDS-CT	**cCTCGAGAAGCTT**GAAAATAAAGTTTGTTATTGGATTTTTGTTAGTTATGGTTGGTGGTTAGGT<br>TTATGTTGAGGTTTAAGATGGTTTATTAGTTAAAGAATGGATGGAAAAGTAGGGAGTATTTGAG<br>TGTGTGATTGATGAGGTGTTTATTGTTATGTTAAAGGTGTTAAATTTTATAAATTTTGATGAATT<br>GTTAATGTAATGTATTTTGATAGTTTTGAATTGGTTTTTTTAGGAAAAATATGGTTTTAAGATGG<br>TATTTTTGGATGGTAATTTTTTGGAAAGGTTTTGTATGTTAGTAGTGGATTATATTTGATTATTA<br>GGTGGGGAAGTGTAATTTAATTTTAGGATAAAGAAAATTGAGTTTAATGATGATGGTATGGTTA<br>AGAGTTTTTTATTTATTAATGGAAGTATTGTTGAAGGAGATGTTTATGTGTTTGTTGTTTTAGTT<br>GATATTTTGAA**GGATCCGGTACCc**
