# Supplementary material for: Nucleotide mismatches prevent intrinsic self-silencing of hpRNA transgenes to enhance RNAi stability in plants
Source: Nat Commun. 2022 Jul 7;13:3926. doi: 10.1038/s41467-022-31641-5 (PMC9263138; doi:10.1038/s41467-022-31641-5)
Supplement: Supplementary file 3 — Description of Additional Supplementary Files [file 41467_2022_31641_MOESM3_ESM.pdf]

Description of Additional Supplementary Files

File Name: Supplementary Data 1
Description: Sequences for McrBC-PCR, bisulfite sequence and RT-PCR primers, 21 and 24-nt GUS sRNA size markers, and oligonucleotides for preparing tri-, di- and mono-phosphorylated EIN2 sRNA
