# Supplementary material for: Nucleotide mismatches prevent intrinsic self-silencing of hpRNA transgenes to enhance RNAi stability in plants
Source: Nat Commun. 2022 Jul 7;13:3926. doi: 10.1038/s41467-022-31641-5 (PMC9263138; doi:10.1038/s41467-022-31641-5)
Supplement: Supplementary file 5 — Reporting Summary [file 41467_2022_31641_MOESM5_ESM.pdf]

nature portfolio

Corresponding author(s): Ming-Bo Wang

Last updated by author(s): 23/6/22

Reporting Summary

Nature Portfolio wishes to improve the reproducibility of the work that we publish. This form provides structure for consistency and transparency in reporting. For further information on Nature Portfolio policies, see our Editorial Policies and the Editorial Policy Checklist.

Statistics

For all statistical analyses, confirm that the following items are present in the figure legend, table legend, main text, or Methods section.

n/a	Confirmed	
☐	☒	The exact sample size (*n*) for each experimental group/condition, given as a discrete number and unit of measurement
☐	☒	A statement on whether measurements were taken from distinct samples or whether the same sample was measured repeatedly
☒	☐	The statistical test(s) used AND whether they are one- or two-sided<br>*Only common tests should be described solely by name; describe more complex techniques in the Methods section.*
☒	☐	A description of all covariates tested
☒	☐	A description of any assumptions or corrections, such as tests of normality and adjustment for multiple comparisons
☐	☒	A full description of the statistical parameters including central tendency (e.g. means) or other basic estimates (e.g. regression coefficient) AND variation (e.g. standard deviation) or associated estimates of uncertainty (e.g. confidence intervals)
☒	☐	For null hypothesis testing, the test statistic (e.g. *F*, *t*, *r*) with confidence intervals, effect sizes, degrees of freedom and *P* value noted<br>*Give P values as exact values whenever suitable.*
☒	☐	For Bayesian analysis, information on the choice of priors and Markov chain Monte Carlo settings
☒	☐	For hierarchical and complex designs, identification of the appropriate level for tests and full reporting of outcomes
☒	☐	Estimates of effect sizes (e.g. Cohen's *d*, Pearson's *r*), indicating how they were calculated

Our web collection on statistics for biologists contains articles on many of the points above.

Software and code

Policy information about availability of computer code

Data collection	No software was used for data collection
Data analysis	ImageJ (http://rsb.info.nih.gov/ij) was used to measure hypocotyl length of hpEIN2 seedlings. For small RNA sequence analysis, Cutadapt version 1.12 (https://cutadapt.readthedocs.io/en/stable/installation.html) was used to trim the adaptor sequences and filter out >35 nt or <18 nt sequences. Bowtie version 1.2.3 (http://bowtie-bio.sourceforge.net/index.shtml) was used to map the clean reads to reference hpEIN2 and hpGUS sequences, without mismatch. Code used in small RNA sequencing analysis is freely available (https://github.com/CSIRO-RNA/size_distribution-of-sRNAs/blob/main/Size_distribution.pl). R version 3.6.0 (2019-04-26) with R studio and BoxPlotR (http://shiny.chemgrid.org/boxplotr/) was used for preparing the boxplots.

For manuscripts utilizing custom algorithms or software that are central to the research but not yet described in published literature, software must be made available to editors and reviewers. We strongly encourage code deposition in a community repository (e.g. GitHub). See the Nature Portfolio guidelines for submitting code & software for further information.

Data

Policy information about availability of data

All manuscripts must include a data availability statement. This statement should provide the following information, where applicable:

- Accession codes, unique identifiers, or web links for publicly available datasets
- A description of any restrictions on data availability
- For clinical datasets or third party data, please ensure that the statement adheres to our policy

All data presented in this article is freely available. The small RNA sequencing data is accessible via GSE178565 (https://www.ncbi.nlm.nih.gov/geo/query/acc.cgi?acc=GSE178565). The source data for all figures and supplementary figures are included in the source data files "Source data for gel blots Main Figures" and "Source

data for MUG-Bisulfite-qPCR", and the "Supplementary Information" file. Nucleotide sequences of all oligonucleotides and primers are available in the "Supplementary Data 1" file and "Supplementary Information".

Field-specific reporting

Please select the one below that is the best fit for your research. If you are not sure, read the appropriate sections before making your selection.

[x] Life sciences [ ] Behavioural & social sciences [ ] Ecological, evolutionary & environmental sciences

For a reference copy of the document with all sections, see nature.com/documents/nr-reporting-summary-flat.pdf

Life sciences study design

All studies must disclose on these points even when the disclosure is negative.

Sample size	Whole transgenic populations, containing large numbers (33-172) of independent plant lines, were used for analyzing GUS and PDS RNAi. Twenty randomly selected independent lines were used for analyzing EIN2 silencing. Independent transgenic lines normally have variations in transgene insertion sites and copy number, so inclusion of multiple (at least two) independent lines is necessary to verify the feature of a particular transgene design. The twenty or more lines are more than sufficient for representing the features of the transgene designs.
Data exclusions	No data exclusions.
Replication	Real time RT-PCR and GUS activity measurement (MUG assay) were performed with three technical replicates for each sample. All attempts at replication were successful.
Randomization	Plant lines for EIN2 RNAi assay were randomly selected. Whole transgenic populations were used for GUS and EIN2 RNAi assay. hpEIN2 and hpGUS lines used for McrBC-PCR methylation analysis were selected based on representative RNAi levels. All RNA and DNA samples from Arabidopsis were isolated from multiple sibling plants for each independent line. Each type of experiments was performed at least twice, either with the same or different sets of transgenic lines for each construct design. Reproducibility of results is ensured by analyzing multiple independent lines, repeating experiments with successful replication of results, and verifying results with two independent technical approaches.
Blinding	N/A (Our study was fully lab based involving no field experiments, and blinding was not found to be required).

Reporting for specific materials, systems and methods

We require information from authors about some types of materials, experimental systems and methods used in many studies. Here, indicate whether each material, system or method listed is relevant to your study. If you are not sure if a list item applies to your research, read the appropriate section before selecting a response.

Materials & experimental systems

n/a	Involved in the study
[x]	[ ] Antibodies
[x]	[ ] Eukaryotic cell lines
[x]	[ ] Palaeontology and archaeology
[x]	[ ] Animals and other organisms
[x]	[ ] Human research participants
[x]	[ ] Clinical data
[x]	[ ] Dual use research of concern

Methods

n/a	Involved in the study
[x]	[ ] ChIP-seq
[x]	[ ] Flow cytometry
[x]	[ ] MRI-based neuroimaging
