# Supplementary material for: Nucleotide mismatches prevent intrinsic self-silencing of hpRNA transgenes to enhance RNAi stability in plants
Source: Nat Commun. 2022 Jul 7;13:3926. doi: 10.1038/s41467-022-31641-5 (PMC9263138; doi:10.1038/s41467-022-31641-5)
Supplement: Supplementary file 6 — Source Data [file 41467_2022_31641_MOESM6_ESM.zip › Source Data/Source data for gel blots.pptx]

## Slide 1
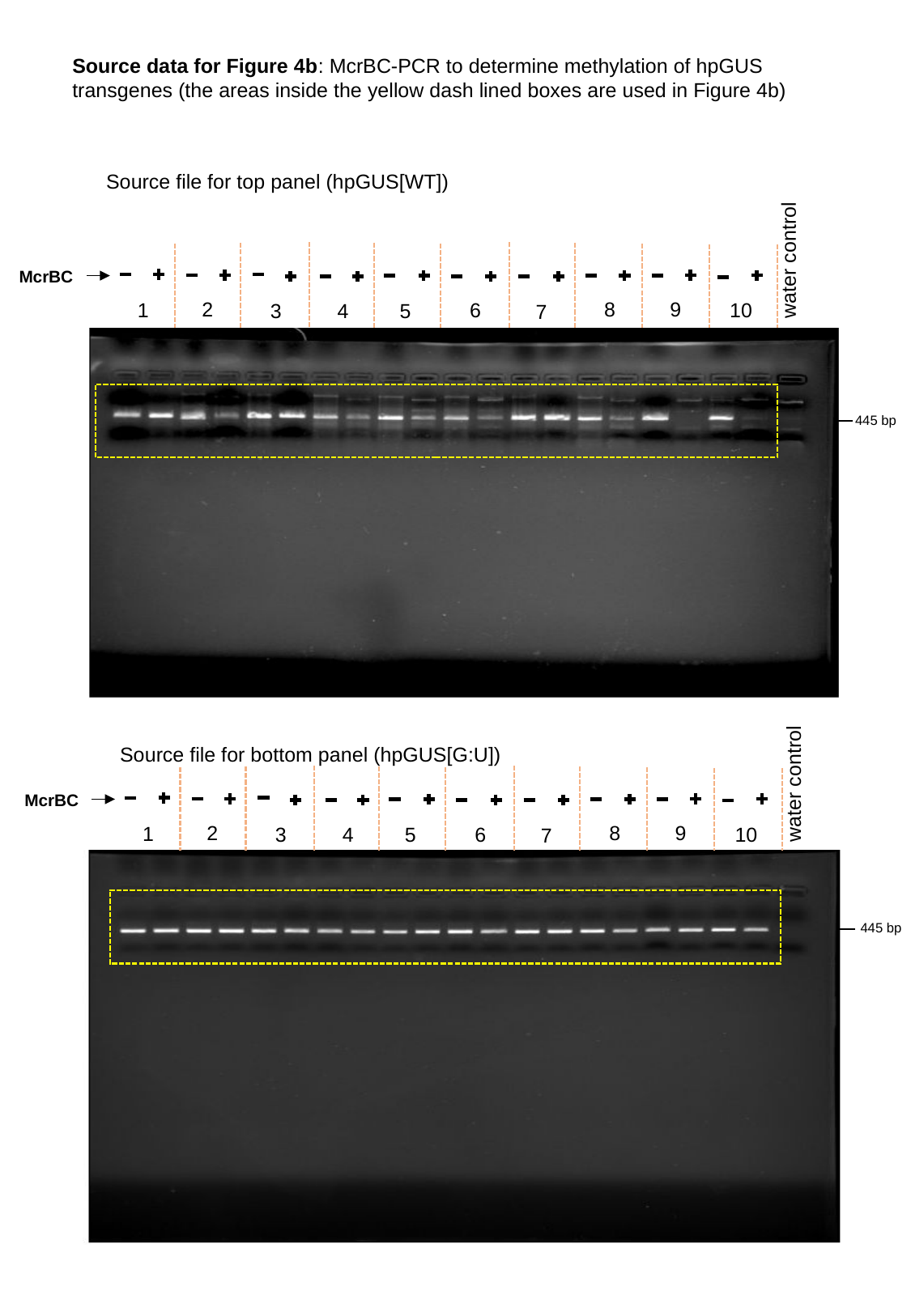

Source data for Figure 4b: McrBC-PCR to determine methylation of hpGUS transgenes (the areas inside the yellow dash lined boxes are used in Figure 4b)
Source file for top panel (hpGUS[WT])
water control
McrBC
2
8
9
1
6
10
4
5
3
7
445 bp
Source file for bottom panel (hpGUS[G:U])
water control
McrBC
2
8
9
1
6
10
4
5
3
7
 445 bp

## Slide 2
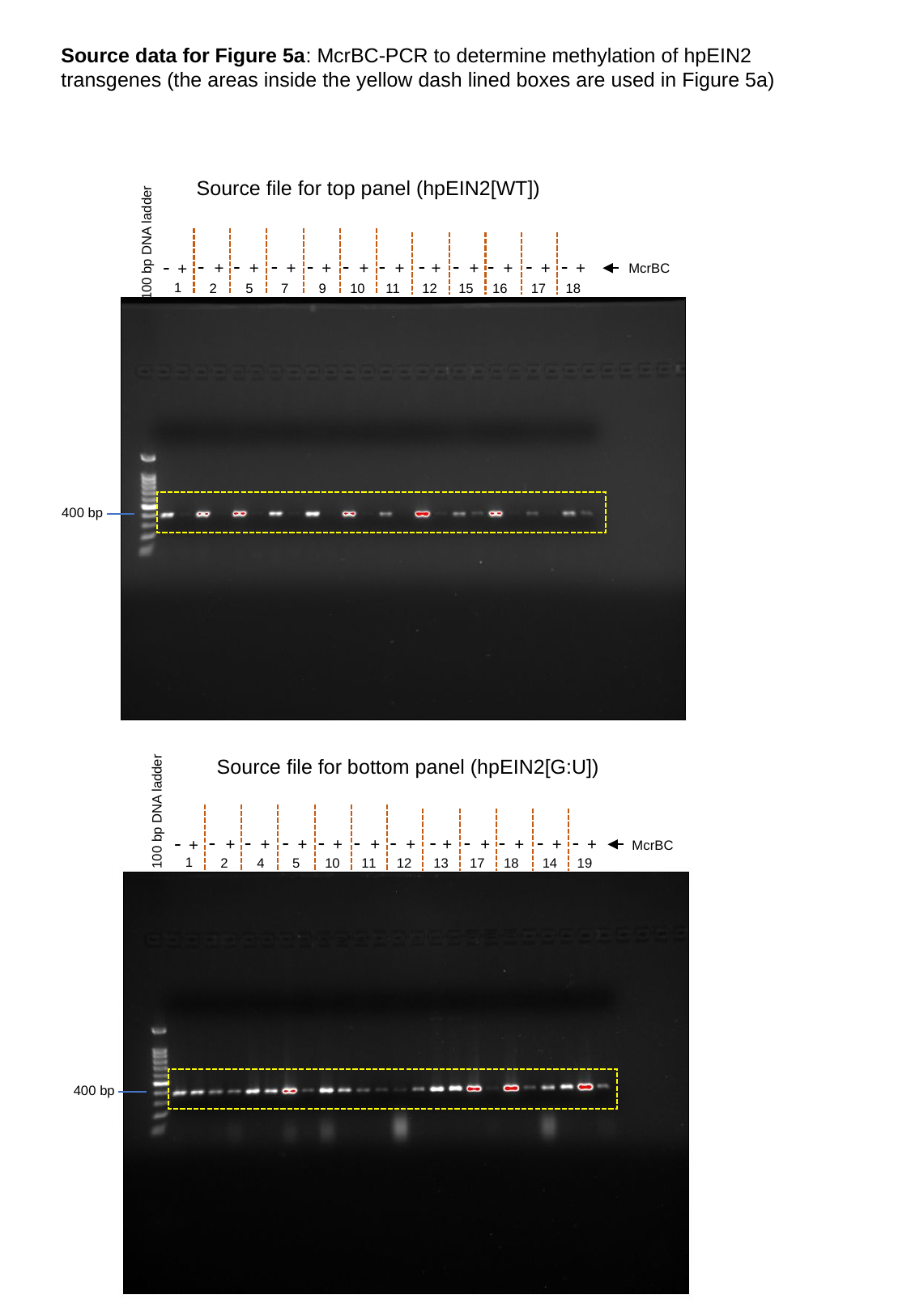

Source data for Figure 5a: McrBC-PCR to determine methylation of hpEIN2 transgenes (the areas inside the yellow dash lined boxes are used in Figure 5a)
Source file for top panel (hpEIN2[WT])
100 bp DNA ladder
-
-
-
-
-
-
-
-
-
-
-
-
+
+
+
+
+
+
+
+
+
+
+
+
McrBC
1
2
5
7
9
10
11
12
15
16
17
18
400 bp
Source file for bottom panel (hpEIN2[G:U])
100 bp DNA ladder
-
-
-
-
-
-
-
-
-
-
-
-
+
+
+
+
+
+
+
+
+
+
+
+
McrBC
1
2
4
5
10
11
12
13
17
18
14
19
400 bp

## Slide 3
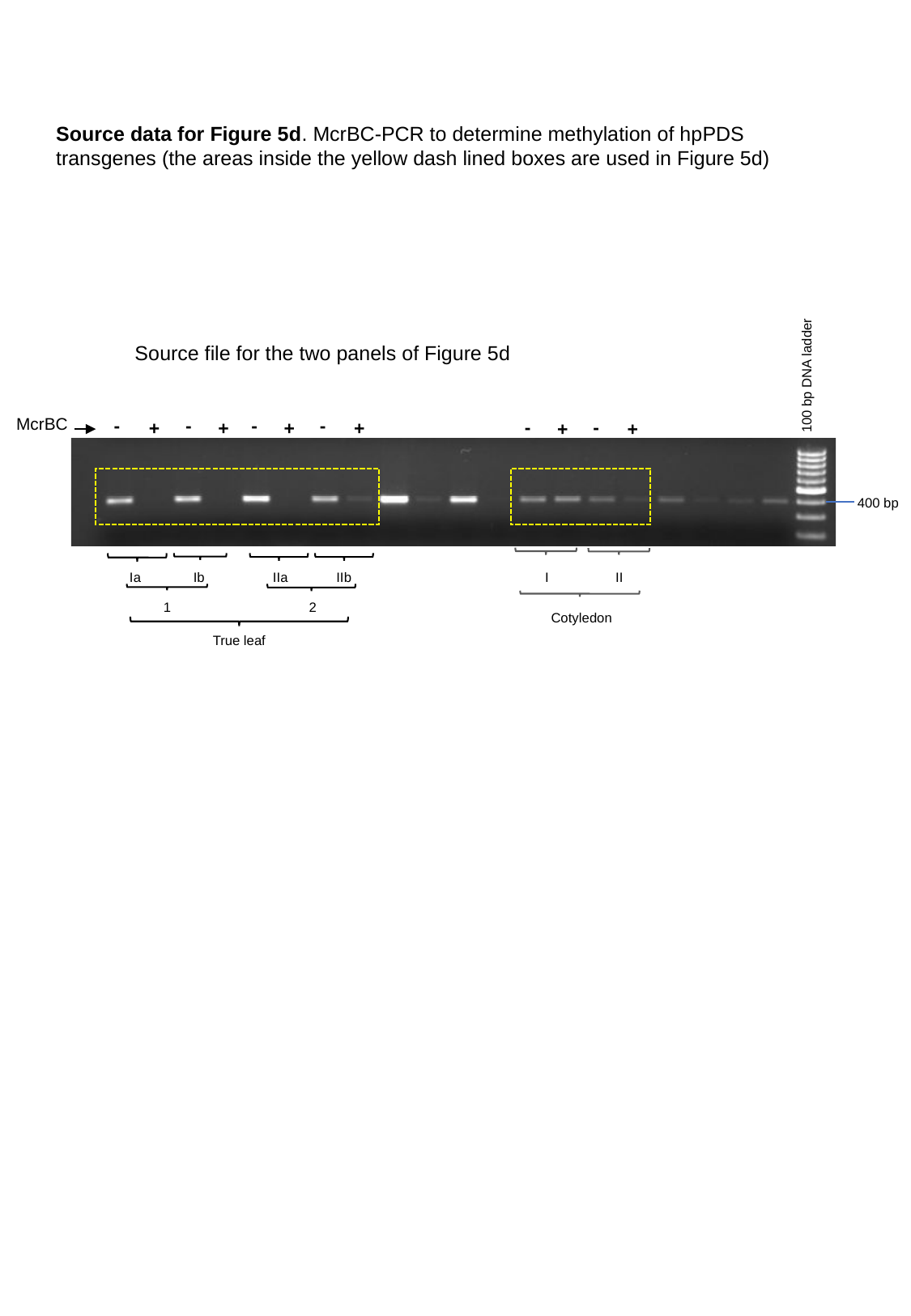

Source data for Figure 5d. McrBC-PCR to determine methylation of hpPDS transgenes (the areas inside the yellow dash lined boxes are used in Figure 5d)
Source file for the two panels of Figure 5d
100 bp DNA ladder
McrBC
-
+
-
+
-
+
-
+
-
+
-
+
400 bp
I
II
Ia
Ib
IIa
IIb
1
2
Cotyledon
True leaf

## Slide 4
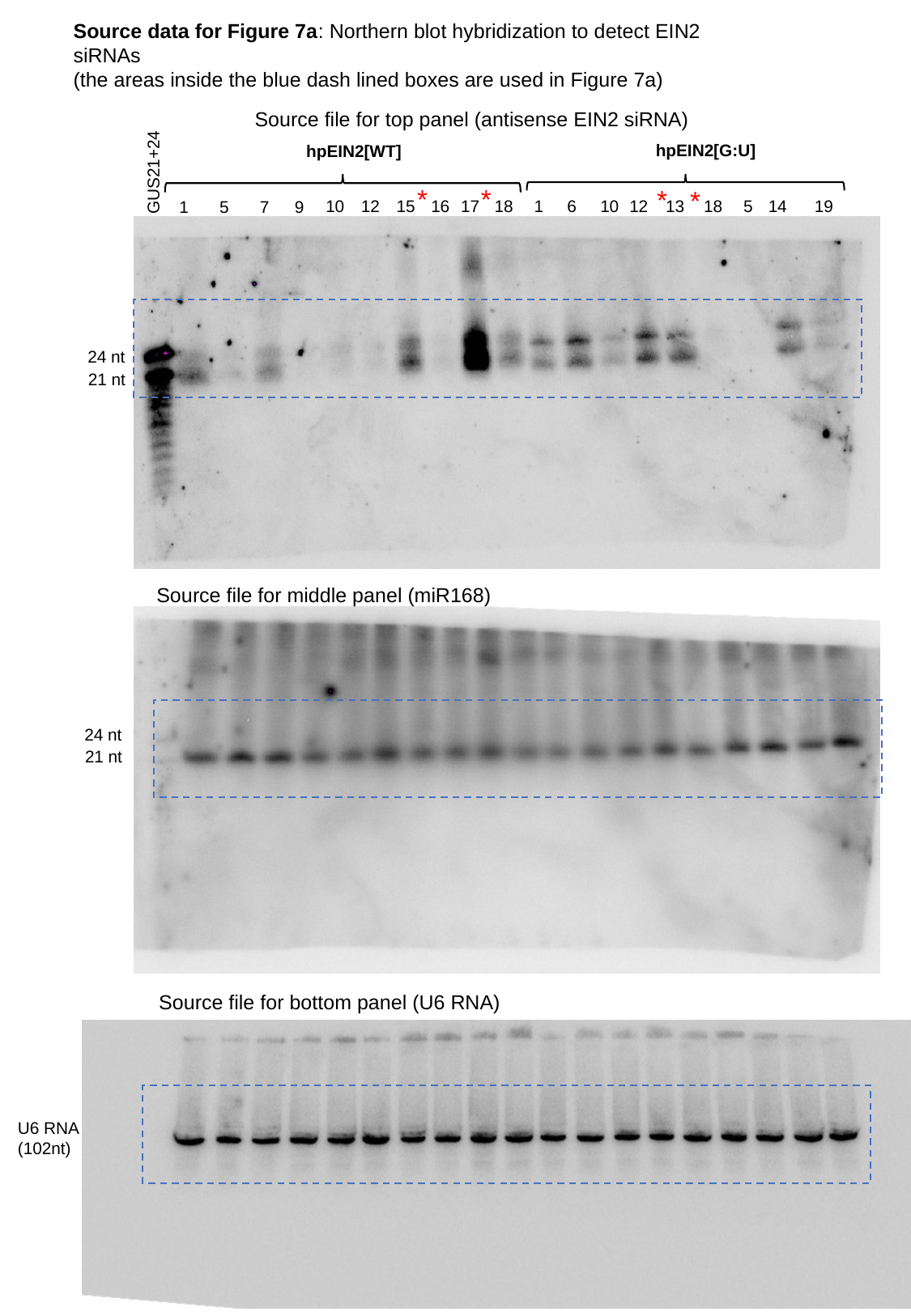

Source data for Figure 7a: Northern blot hybridization to detect EIN2 siRNAs
(the areas inside the blue dash lined boxes are used in Figure 7a)
Source file for top panel (antisense EIN2 siRNA)
hpEIN2[WT]
hpEIN2[G:U]
GUS21+24
*
*
*
*
10
15
16
12
17
1
6
10
18
13
18
12
5
19
14
1
7
9
5
24 nt
21 nt
Source file for middle panel (miR168)
24 nt
21 nt
Source file for bottom panel (U6 RNA)
U6 RNA
(102nt)

## Slide 5
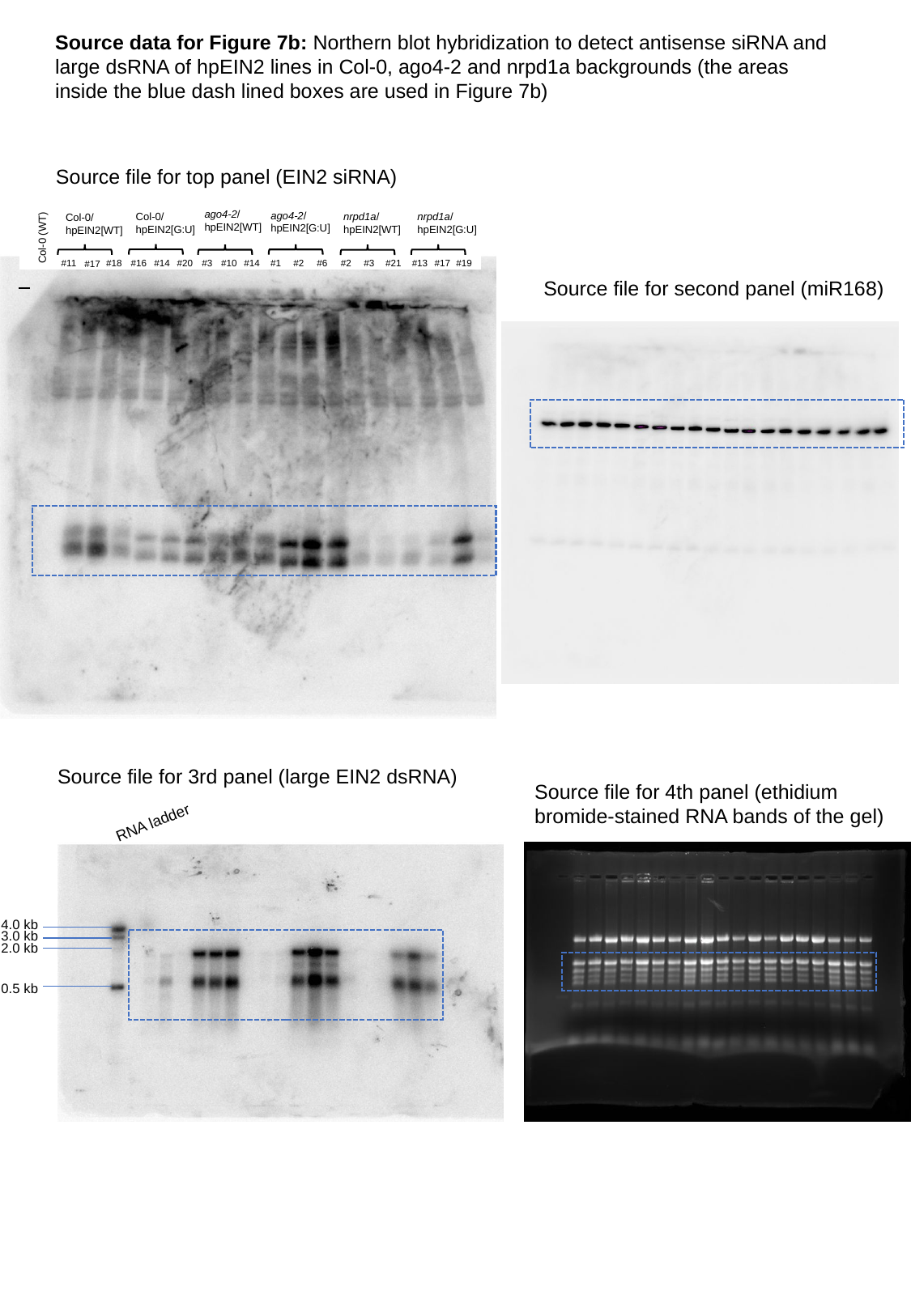

Source data for Figure 7b: Northern blot hybridization to detect antisense siRNA and large dsRNA of hpEIN2 lines in Col-0, ago4-2 and nrpd1a backgrounds (the areas inside the blue dash lined boxes are used in Figure 7b)
Source file for top panel (EIN2 siRNA)
ago4-2/
hpEIN2[WT]
ago4-2/
hpEIN2[G:U]
Col-0/
hpEIN2[G:U]
nrpd1a/
hpEIN2[G:U]
nrpd1a/
hpEIN2[WT]
Col-0/
hpEIN2[WT]
b
Col-0 (WT)
#2
#10
#18
#3
#17
#20
#6
#14
#11
#21
#19
#16
#1
#3
#2
#13
#14
#17
Source file for second panel (miR168)
Source file for 3rd panel (large EIN2 dsRNA)
Source file for 4th panel (ethidium bromide-stained RNA bands of the gel)
RNA ladder
4.0 kb
3.0 kb
2.0 kb
0.5 kb

## Slide 6
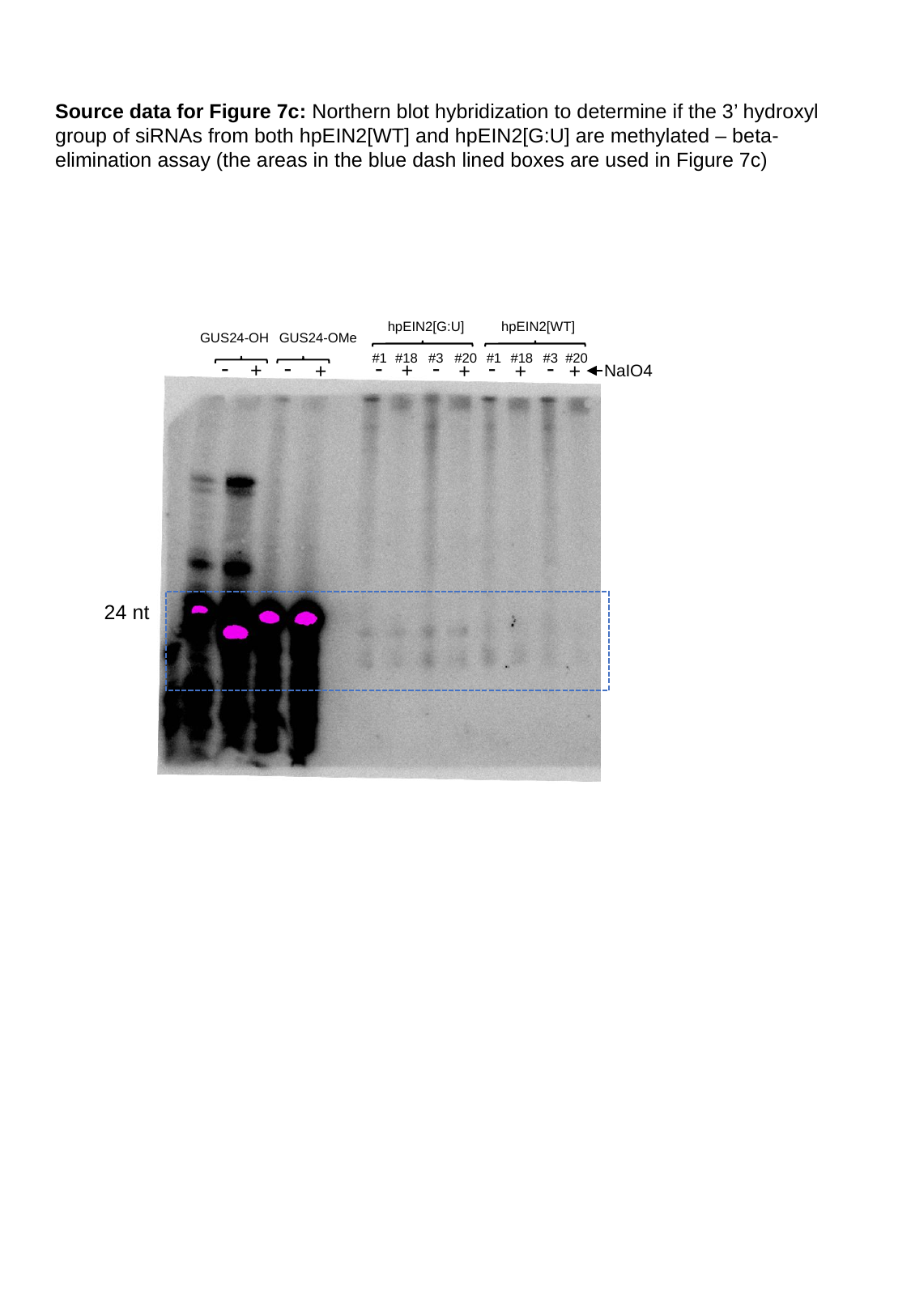

Source data for Figure 7c: Northern blot hybridization to determine if the 3’ hydroxyl group of siRNAs from both hpEIN2[WT] and hpEIN2[G:U] are methylated – beta-elimination assay (the areas in the blue dash lined boxes are used in Figure 7c)
hpEIN2[WT]
hpEIN2[G:U]
GUS24-OH
GUS24-OMe
#1
#18
#3
#20
#1
#18
#3
#20
-
-
-
-
-
-
+
+
+
+
+
+
NaIO4
24 nt

## Slide 7
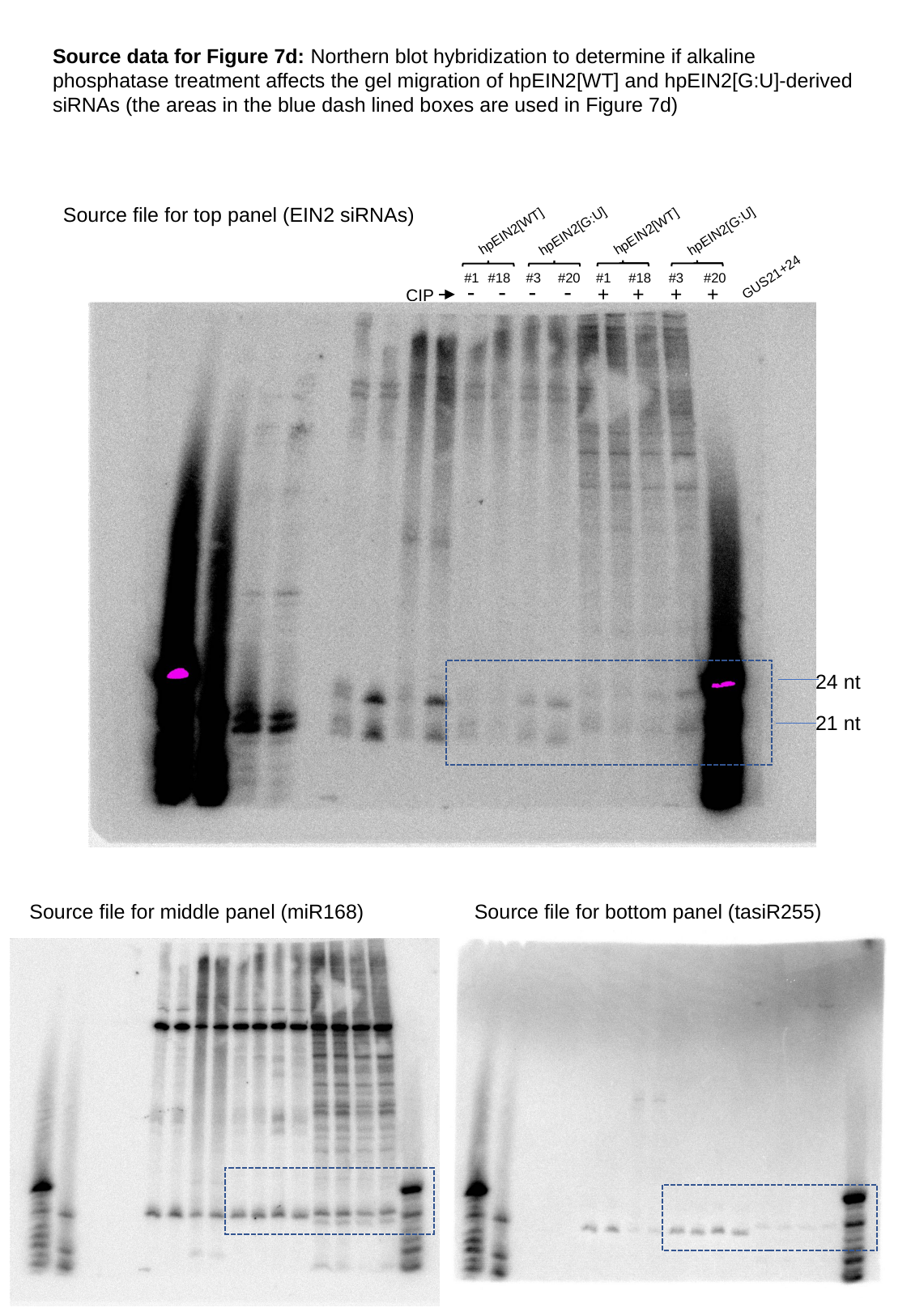

Source data for Figure 7d: Northern blot hybridization to determine if alkaline phosphatase treatment affects the gel migration of hpEIN2[WT] and hpEIN2[G:U]-derived siRNAs (the areas in the blue dash lined boxes are used in Figure 7d)
Source file for top panel (EIN2 siRNAs)
hpEIN2[G:U]
hpEIN2[G:U]
hpEIN2[WT]
hpEIN2[WT]
GUS21+24
#1
#18
#3
#20
#1
#18
#3
#20
-
-
-
-
+
+
+
+
CIP
24 nt
21 nt
24 nt
21 nt
Source file for middle panel (miR168)
Source file for bottom panel (tasiR255)

## Slide 8
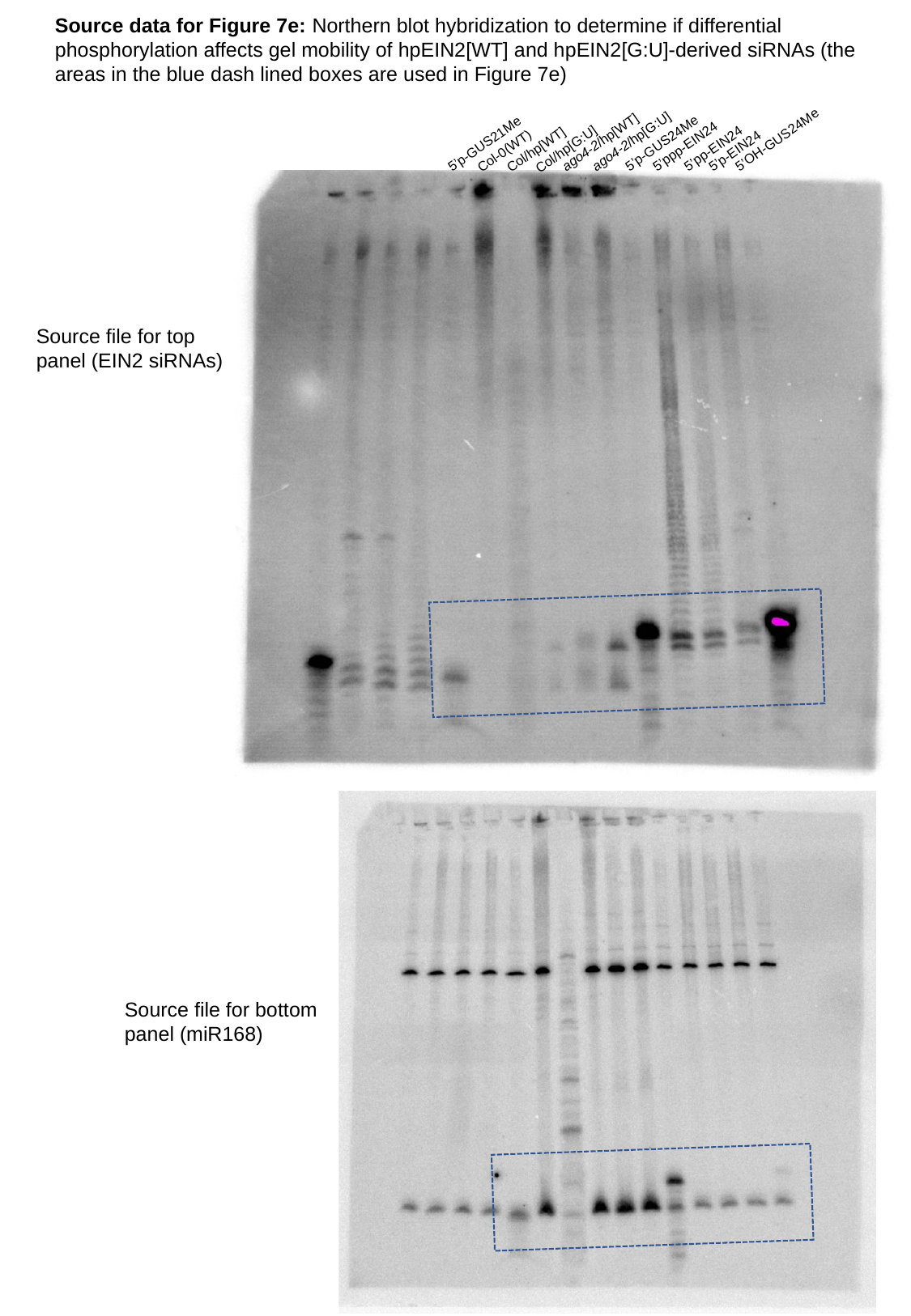

Source data for Figure 7e: Northern blot hybridization to determine if differential phosphorylation affects gel mobility of hpEIN2[WT] and hpEIN2[G:U]-derived siRNAs (the areas in the blue dash lined boxes are used in Figure 7e)
5’OH-GUS24Me
5’ppp-EIN24
5’p-GUS21Me
ago4-2/hp[G:U]
ago4-2/hp[WT]
5’p-GUS24Me
5’p-EIN24
5’pp-EIN24
Col/hp[G:U]
Col/hp[WT]
Col-0(WT)
Source file for top panel (EIN2 siRNAs)
Source file for bottom panel (miR168)

## Slide 9
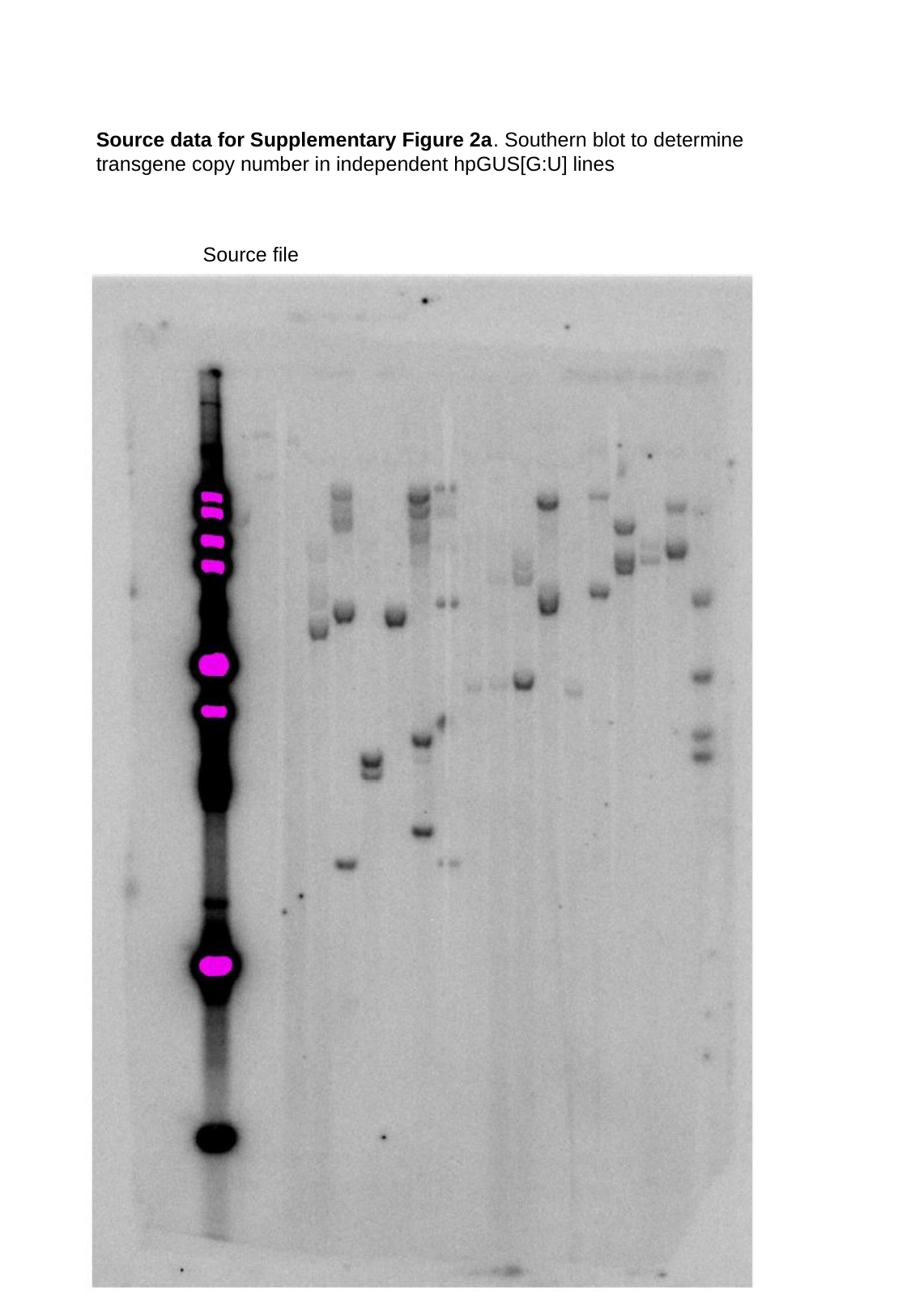

Source data for Supplementary Figure 2a. Southern blot to determine transgene copy number in independent hpGUS[G:U] lines
Source file

## Slide 10
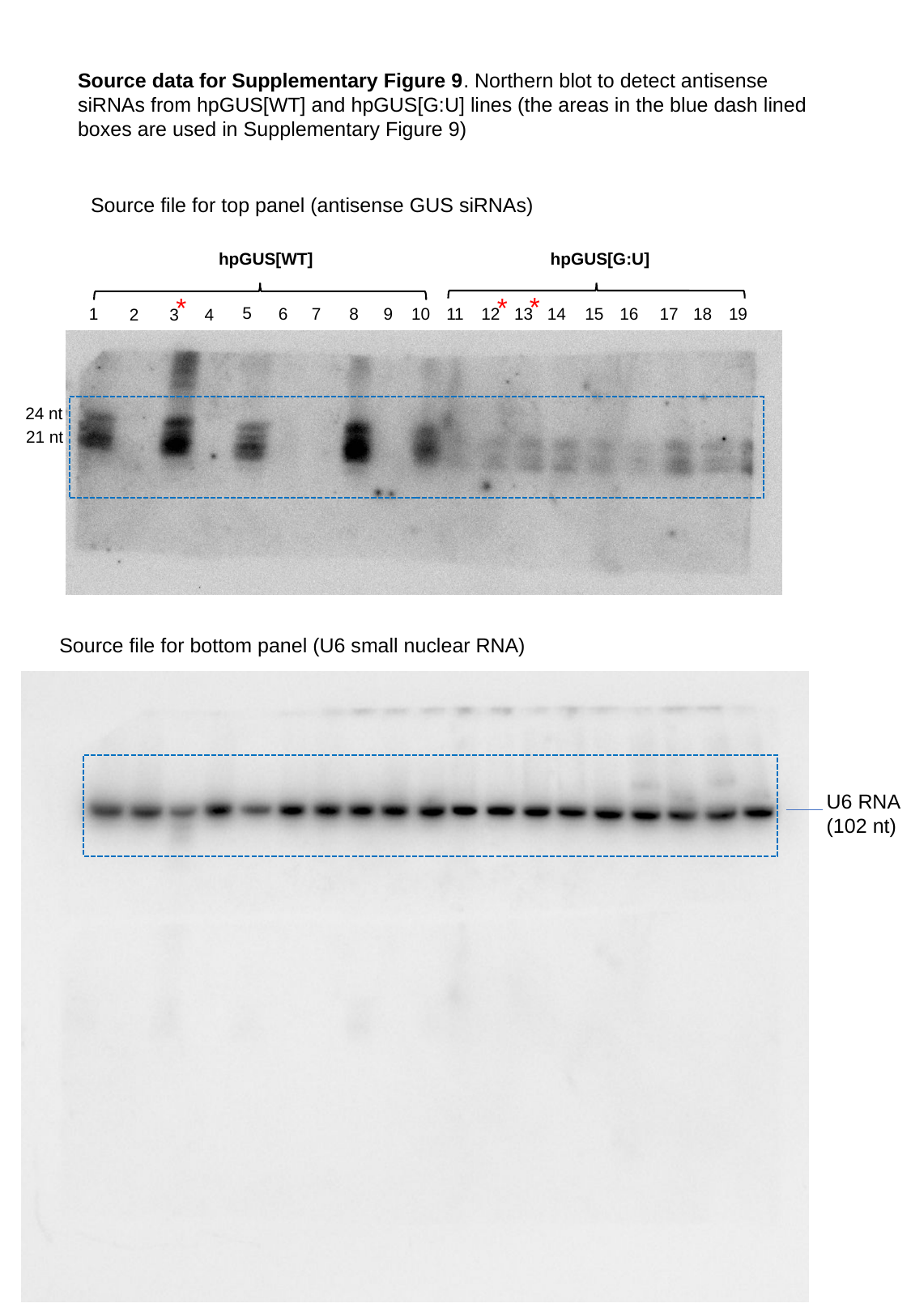

Source data for Supplementary Figure 9. Northern blot to detect antisense siRNAs from hpGUS[WT] and hpGUS[G:U] lines (the areas in the blue dash lined boxes are used in Supplementary Figure 9)
Source file for top panel (antisense GUS siRNAs)
hpGUS[WT]
hpGUS[G:U]
*
*
*
5
7
8
6
9
11
12
13
10
15
16
14
17
19
18
1
3
4
2
24 nt
21 nt
Source file for bottom panel (U6 small nuclear RNA)
U6 RNA
(102 nt)

## Slide 11
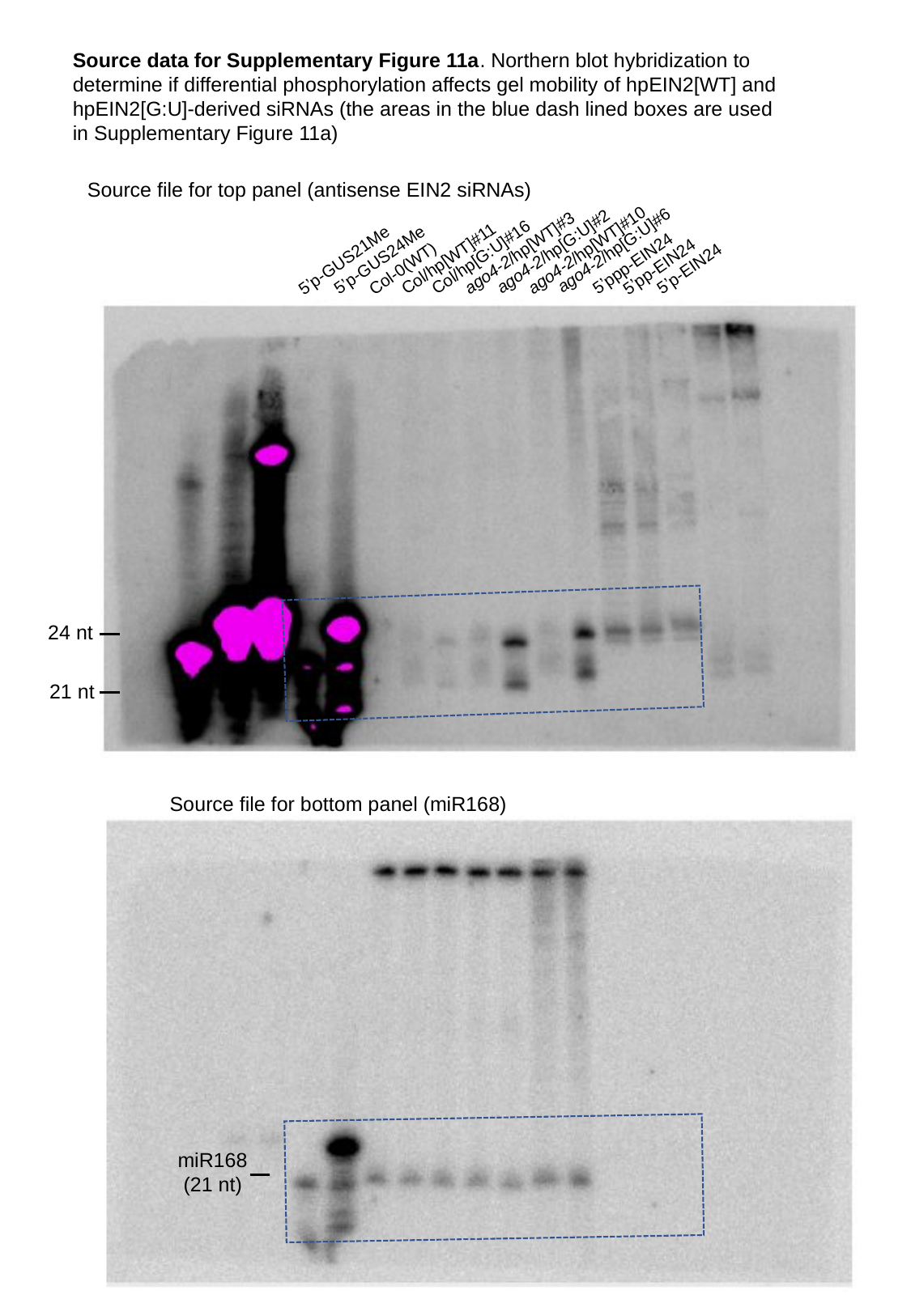

Source data for Supplementary Figure 11a. Northern blot hybridization to determine if differential phosphorylation affects gel mobility of hpEIN2[WT] and hpEIN2[G:U]-derived siRNAs (the areas in the blue dash lined boxes are used in Supplementary Figure 11a)
Source file for top panel (antisense EIN2 siRNAs)
5’p-GUS21Me
ago4-2/hp[WT]#10
ago4-2/hp[G:U]#6
5’ppp-EIN24
ago4-2/hp[G:U]#2
5’p-GUS24Me
ago4-2/hp[WT]#3
5’p-EIN24
5’pp-EIN24
Col/hp[G:U]#16
Col/hp[WT]#11
Col-0(WT)
24 nt
21 nt
Source file for bottom panel (miR168)
miR168
 (21 nt)

## Slide 12
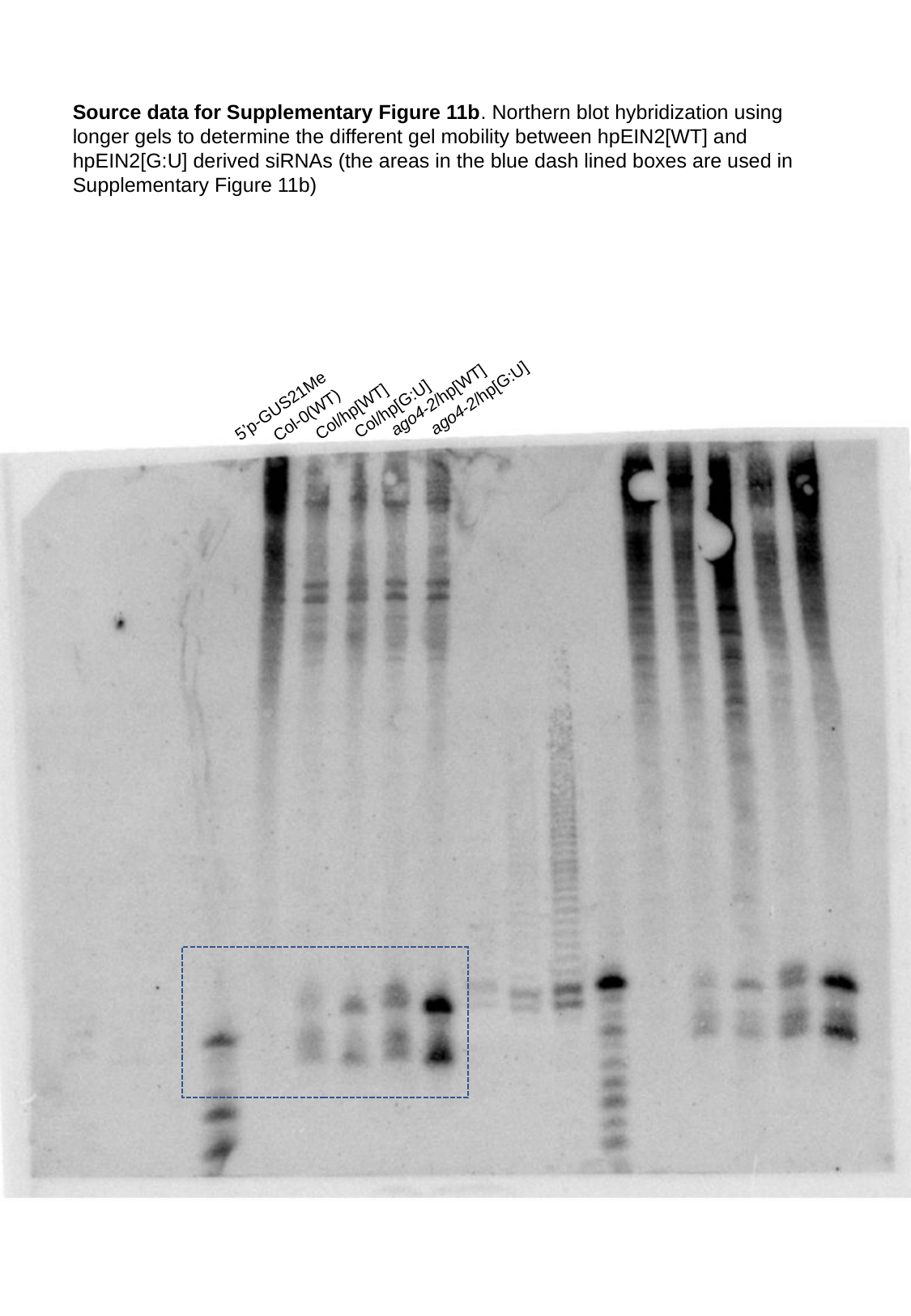

Source data for Supplementary Figure 11b. Northern blot hybridization using longer gels to determine the different gel mobility between hpEIN2[WT] and hpEIN2[G:U] derived siRNAs (the areas in the blue dash lined boxes are used in Supplementary Figure 11b)
5’p-GUS21Me
ago4-2/hp[G:U]
ago4-2/hp[WT]
Col/hp[G:U]
Col/hp[WT]
Col-0(WT)
